# Mitoribosomal regulation of OXPHOS biogenesis in plants

**DOI:** 10.3389/fpls.2014.00079

**Published:** 2014-03-05

**Authors:** Hanna Janska, Malgorzata Kwasniak

**Affiliations:** Molecular Biology of the Cell Department, Faculty of Biotechnology, University of WroclawWroclaw, Poland

**Keywords:** OXPHOS, mitoribosome, mitochondrial translation, ribosome filter hypothesis, ribosome heterogeneity, coordination of gene expression

## Abstract

The ribosome filter hypothesis posits that ribosomes are not simple non-selective translation machines but may also function as regulatory elements in protein synthesis. Recent data supporting ribosomal filtering come from plant mitochondria where it has been shown that translation of mitochondrial transcripts encoding components of oxidative phosphorylation complexes (OXPHOS) and of mitoribosomes can be differentially affected by alterations in mitoribosomes. The biogenesis of mitoribosome was perturbed by silencing of a gene encoding a small-subunit protein of the mitoribosome in *Arabidopsis thaliana*. As a consequence, the mitochondrial OXPHOS and ribosomal transcripts were both upregulated, but only the ribosomal proteins were oversynthesized, while the OXPHOS subunits were actually depleted. This finding implies that the heterogeneity of plant mitoribosomes found *in vivo* could contribute to the functional selectivity of translation under distinct conditions. Furthermore, global analysis indicates that biogenesis of OXPHOS complexes in plants can be regulated at different levels of mitochondrial and nuclear gene expression, however, the ultimate coordination of genome expression occurs at the complex assembly level.

## INTRODUCTION

It is a widely held belief that post-transcriptional control dominates in the regulation of mitochondrial gene expression in plants, but the experimental evidence was until recently limited to events affecting RNA quality and quantity (Binder and Brennicke, 2003). Translational regulation in plant mitochondria was expected but, until now (Kwaśniak et al., 2013), only hypothetical. The basic process of translation involves the decoding of the mRNA-encoded information into proteins by ribosomes. Therefore, historically, ribosomes were considered to have a constitutive rather than a regulatory function, and the efficiency of translation was believed to be determined either by features of the mRNA itself or by mRNA-binding factors. However, a number of observations indicated that ribosomes themselves could also differentially affect the translation of particular mRNAs (Mauro and Edelman, 2007). This mini-review summarizes current knowledge on ribosomal regulation of OXPHOS biogenesis.

## RIBOSOME HETEROGENEITY AS BASIS FOR RIBOSOME FILTER HYPOTHESIS

All ribosomes consist of the large subunit (LSU) and small subunit (SSU) composed of both proteins and rRNAs. Originally, ribosomes of an organism were viewed as homogeneous entities showing no variation in their rRNA or protein composition despite the observation of a growth rate-dependent ribosome heterogeneity in *Escherichia coli* made over 40 years ago (Deusser, 1972). The earliest evidence demonstrating heterogeneity of ribosomes in a eukaryotic cell emerged in 1981 during studies on the social amoeba *Dictyostelium discoideum *(Ramagopal and Ennis, 1981). The ribosomes from the different developmental stages of *D. discoideum* varied in their *composition and* covalent modifications (Ramagopal, 1992). Since then ample evidence has accumulated supporting the view that heterogeneous pools of ribosomes exhibiting variations in the RNA and or the protein components are present in bacteria and in eukaryotic cells (Brygazov et al., 2013; Filipovska and Rackham, 2013). Since the focus of this review is the translational regulation in plant mitochondria the heterogeneity of plant mitoribosomes will be presented in more detail.

The plant mitoribosome contains three rRNA molecules encoded in the mitochondrial DNA, 26S and 5S for the LSU, and 18S for the SSU. The rRNAs undergo several important post-transcriptional modifications (pseudouridylation and methylation; Bonen, 2004), and an rRNA methyltransferase required for the dimethylation of two conserved adenines in the mitochondrial 18S rRNA of *Arabidopsis *was characterized (Richter et al., 2010). In contrast to the rRNAs, the mitoribosomal proteins are encoded by both the mitochondrial and nuclear genomes. The sets of proteins encoded by these two genomes vary between plant species (Salinas et al., 2013), and the exact protein composition of plant mitoribosomes has not been fully determined yet. A recent bioinformatics analysis identified 71 genes encoding mitoribosomal proteins in *A. thaliana*, among them eight in the mitochondrial genome (Sormani et al., 2011). It should be emphasized that these numbers do not represent unique genes, since 16 of mitochondrial ribosomal proteins are encoded by more than one copy of gene. Potato and broad bean proteomics found 68–80 mitoribosomal proteins (Pinel et al., 1986; Maffey et al., 1997). Thus, compared with the bacterial ribosomes with only 54 proteins (Wittmann, 1982), the plant mitoribosome is apparently more complex. The plant mitochondrial ribosomal proteins are of two evolutionarily distinct types, those with a direct bacterial origin and those recruited during the evolution (Salinas et al., 2013). Additionally, in *Arabidopsis* two proteins have been identified to be associated with mitoribosomes, namely PNM1 and PPR336 (Uyttewaal et al., 2008; Hammani et al., 2011). They belong to the pentatricopeptide repeat (PPR) protein family that is particularly large in higher plants, but their role in mitochondrial translation is still unknown.

The first indication of a possible heterogeneity of plant mitoribosomes comes from a study on four paralogs of ribosomal protein L12 in potato (Delage et al., 2007). At the RNA level these paralogs are expressed simultaneously and at similar abundance. All four L12 variants were present in the mitochondrial ribosome fraction, but they showed a divergent tendency to dissociate upon treatments that affects ribosomes integrity. The presence of the four paralogs of slightly different proportion suggests either occurrence of heterogeneous L12 composition among each mitoribosome and/or a heterogeneous population of mitochondrial ribosomes in the plant cell.

A heterogeneity of plant mitoribosomes is also implied by several developmental phenotypes of mutants defective in a mitoribosomal protein (Schippers and Mueller-Roeber, 2010). Slightly different phenotypes connected with defects in leaf morphology have been reported for three *Arabidopsis* mutants. In all three, the leaves are smaller, but an irregular leaf shape is characteristic for the mutant with reduced expression of both *MRPS3* and *MRPL16* (Sakamoto et al., 1996) as well as the mutant with RNAi-dependent silencing of *MRPS10* (Majewski et al., 2009), but not for the mutant with a 90% reduction in *MRPL11* transcript (Pesaresi et al., 2006). The alteration in leaf morphology was observed also in maize *rps3-rpl16* mutants, which produces severely stunted plants with striations on the leaves (Hunt and Newton, 1991). The role of mitoribosomal proteins in other specific developmental processes is underlined by several specific *Arabidopsis* mutants with defects in genes: *MRPL14*, which is essential for ovule development (Schneitz et al., 1998; Skinner et al., 2001), *MRPL21* and *MRPS11* which are required during female gametophyte development (Portereiko et al., 2006), as well as *MRPS16 *gene, in which transposon-induced knockout causes an embryo-defective lethal phenotype (Tsugeki et al., 1996). Thus, specific mitoribosomal proteins appear to influence selectively different phases of the plant development, hinting on the existence of specialized ribosomes with the translational activity critical at specific developmental stages. This view is strengthened by the finding that the expression of individual *A. thaliana *genes encoding mitochondrial ribosomal proteins is highly variable during leaf development (Schippers and Mueller-Roeber, 2010). A functional heterogeneity of ribosomes could also be generated by interactions with accessory proteins as well as posttranslational modifications of ribosomal proteins, but the significance, if any of these factors for plant mitoribosomes is unknown.

The apparent ribosome heterogeneity formed a basis for the ribosome filter hypothesis in which the ribosome acts like a filter selecting specific mRNAs and consequently modulating translation (Mauro and Edelman, 2007). The different subpopulations of ribosomes would vary in their ability to translate specific subsets of mRNA and thus the ribosomes would selectively control translation and, consequently, gene expression. In accordance with the ribosome filter hypothesis, it has recently been shown that the biogenesis of OXPHOS complexes in *A. thaliana *mitochondria is subject to translational control by mitoribosomes (Kwaśniak et al., 2013).

## MITORIBOSOME STATUS SELECTIVELY AFFECTS EFFICIENCY OF TRANSLATION OF MITOCHONDRIAL mRNAs FOR OXPHOS AND RIBOSOMAL PROTEINS IN *Arabidopsis*

Mitochondrial translation is indispensable for the biogenesis of the OXPHOS machinery and mitoribosomes simply because several protein subunits of the both types of complexes are synthesized in mitochondria. Spice has been added to that simple story by recent data indicating that plant mitoribosomes do not synthesize proteins encoded in the mitochondrial genome non-selectively but rather execute a transcript-specific translational control (Kwaśniak et al., 2013).

**FIGURE 1:**
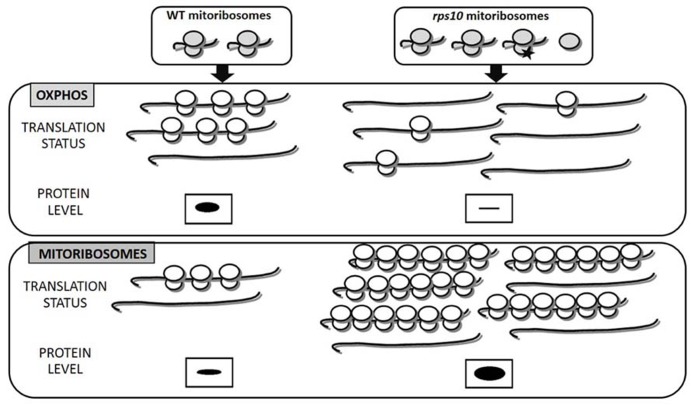
**Heterogeneity of mitoribosomes differentially affects the translation of mitochondrial OXPHOS and ribosomal proteins (based on Kwaśniak et al., 2013).** In *rps10* protein synthesis is carried out by a heterogeneous population of mitoribosomes characterized by an excess of free LSU subunits and a portion of SSUs lacking the S10 protein (black asterisk). Decreased translation of OXPHOS in *rps10* results mainly from the reduced number of ribosomes per mRNA, whereas increased translation of mitoribosomal proteins results from enhanced ribosomal loading and also increased ribosomal density. In consequence, OXPHOS proteins are depleted, whereas mitoribosomal proteins over-abundant in *rps10* mitochondria compared to wild-type ones. The amount of proteins translated by mitoribosomes is represented by the size of the black bands.

This finding is fully consistent with the ribosome filter theory. RNAi-mediated silencing leads to the generation of unique, heterogeneous population of ribosomes. Ribosomes lacking the S10 protein coexisted with wild-type ones and an excess of free LSU (**Figure 1**). It should be underlined that the *rps10* mutant did not suffer from an insufficiency of wild-type mitoribosomes since the silencing provoked a compensatory response increasing the overall abundance of both small and LSUs. The polymorphic population of mitoribosomes turned out to translate two subsets of mRNAs, those encoding OXPHOS subunits and ribosomal proteins, with altered efficiency compared to the wild-type. The majority of the OXPHOS transcripts were translated less efficiently, whereas most of the mitoribosome protein transcripts were translated with an enhanced efficiency relative to the wild-type. It should be emphasized that in the *RPS10*-silenced plants the mitochondrial transcripts for both OXPHOS and ribosomal proteins were up-regulated. Thus, the altered translation was not correlated with the transcript level but was due to an altered efficiency of their binding by the mitoribosomes. As a consequence, the rate of synthesis of the OXPHOS proteins was below that observed in wild-type mitochondria despite the over-abundance of their mRNAs. Thus, the diversified mitochondrial translation efficiency modulated profoundly the outcome of the earlier steps of mitochondrial genome expression. The effect of the altered SSU and/or the excess of free LSU on the efficiency of protein synthesis in the *rps10* mitochondria was postulated to have resulted from the formation of unique interactions between those subunits and other components of the translation apparatus. It is tempting to speculate that similar translational regulation could occur in wild-type plants, namely under conditions that lead to heterogeneity of mitoribosome population in response to environmental or developmental signals.

According to existing reports plant mitochondrial RNAs have long 5′ untranslated regions (UTR), which could be involved in translational efficiency by interacting with altered SSU and/or the excess of free LSU. However, no obvious motifs have been found within the 5′ UTR sequences suggesting that these parts of mRNAs are involved in gene-specific translation regulation (Kazama et al., 2013), whereas in *rps10 *rather group-specific translational regulation was observed. Future studies are awaited to understand how differential translation of OXPHOS and mitoribosomal proteins is achieved in the *rps10* mutant.

The importance of mitochondrial translational regulation for OXPHOS complex I biogenesis has recently been suggested basing on analysis of an *A. thaliana* mutant with a depletion of the mitochondrial matrix iron-sulfur protein required for NADH dehydrogenase (INDH)(Wydro et al., 2013). INDH was shown not to be a complex I-specific translational regulator but rather to have a more general role in mitochondrial translation. Depending on the *A. thaliana *growth stage and the extent of INDH depletion diverse alterations in the mitochondrial translation rate and/or protein pattern were observed in the *indh* mutants. A strong depletion of INDH decreased the rate of mitochondrial translation, whereas the steady state levels of almost all mitochondrial transcripts examined were unaltered or increased.

The data indicating a heterogeneity of plant mitoribosomes combined with the finding that translation could be the overriding regulatory stage of mitochondrial gene expression regulation suggest that in plants the status of the mitoribosomal population could be an essential regulator of expression of mitochondrially encoded proteins in response to developmental or environmental signals. The postulated ribosome-mediated selectivity of mitochondrial translation should be reflected by a poor correlation between the steady-state abundance of mitochondrial transcripts and the encoded proteins. However, despite the recent intense studies of the plant mitochondrial transcriptome and proteome no enough quantitative data exist to substantiate such a claim. Relations between the transcript and the protein level are mainly known for the nuclear-encoded mitochondrial proteins (Law et al., 2012; Tan et al., 2012).

## EXPRESSION OF NUCLEAR AND MITOCHONDRIAL GENES ENCODING OXPHOS SUBUNITS IS ULTIMATELY COORDINATED AT THE COMPLEX ASSEMBLY STAGE

Biogenesis of OXPHOS complexes requires balanced production of nuclear- and mitochondrially encoded subunits. Some reports indicate that in plants coordination of the expression of OXPHOS genes between the nuclear and mitochondrial genomes may occur at the transcript level (Topping and Leaver, 1990; Smart et al., 1994; Ribichich et al., 2001; Howell et al., 2006), but recent global analyses of expression of the mitochondrial and nuclear genomes in plants underscore the crucial role of the post-transcriptional coordination (Giegé et al., 2005; Leister et al., 2011; Law et al., 2012; Kwaśniak et al., 2013). Furthermore, Giegé et al. (2005) and Kwaśniak et al. (2013) studying biogenesis of OXPHOS complexes under sugar starvation conditions and in the *rps10* mutant, respectively, reached the same conclusion that the ultimate coordination of expression of the two genomes occurs at the level of protein complex assembly. The authors of both those papers assumed that an excess of unassembled subunits were degraded by nuclear-encoded ATP-dependent proteases. Apart from this similarity, there were also differences between the two experimental systems, as the *RPS10* silencing mainly down-regulated the biosynthesis of the mitochondrially encoded subunits whereas under sugar starvation the biogenesis of OXPHOS complexes was regulated mostly by curbing the expression of the respective nuclear genes. As a consequence, the nuclear-encoded proteins in *rps10* and the mitochondrially encoded proteins under sugar starvation had to be degraded to bring their abundance down to the level of their partners. One is tempted to suggest that depending on the nature or the duration of the stress the direct response of the two collaborating genomes is in fact limited to one or the other and the expression of the second genome is adjusted ultimately at the complex assembly level.

In line with the above predictions, an up-regulation of mitochondrial ATP-dependent proteases was indeed observed in the *rps10* mutant. An induction of nuclear-encoded mitochondrial proteases and chaperones upon accumulation of unfolded or misfolded proteins in the mitochondrial matrix is a hallmark of the mitochondrial unfolded-protein response (UPR^mt^) that has been reported for mammals and *Caernohabditis elegans* (Haynes and Ron, 2010; Haynes et al., 2013). Conceivably, also in plants UPR^mt^ is activated by the stoichiometric imbalance between the nDNA- and mtDNA-encoded OXPHOS proteins caused by the silencing of *RPS10* expression and restores the balance between the two sets of the subunits by selectively degrading the ones in excess.

## MITOCHONDRIAL TRANSLATION REGULATION OF OXPHOS BIOGENESIS IN NON-PLANT SYSTEMS

In contrast to plants, where translational regulation has just emerged as an important step of the biogenesis of OXPHOS complexes, a diversity of mitochondrial translation control mechanisms have been reported in other organisms, particularly in *Saccharomyces cerevisiae*. One such mechanism is associated with translational activators which interact with mitochondrial mRNAs, in most cases at their 5′ UTR, to facilitate their translation (Smits et al., 2010). It is unclear whether all translational activators bind the mRNA directly or whether some of them interact with other components of the translation machinery (Herrmann et al., 2013). Ample data show that the translational activators can be part of a feedback mechanism coupling the rate of synthesis of mitochondrial proteins to the rate of their assembly into complexes, known as *control by epistasy of synthesis* (CES). This mechanism was first reported for chloroplasts (Choquet and Wollman, 2002). In the yeast mitochondria the best known such feedback loop is the one that controls the synthesis of Cox1. This regulation is achieved by the translational activator Mss51 which interacts with the *Cox1* mRNA, newly synthesized unassembled Cox1 protein, and other Cox assembly factors during Cox1 maturation/assembly (Fontanesi et al., 2010). Assembly defects lead to a sequestration of Mss51 and consequent, stoppage of Cox1 synthesis. A link between mitochondrial translation and complex assembly is also supported by another regulatory mechanism found in yeast, in which generation of assembly competent proteins during mitochondrial translation requires the mitoribosomal protein MPRL36 (Prestele et al., 2009). Mitoribosomes with a C-terminal domain-deficient MRPL36 protein still carry out translation, but the resulting polypeptides fail to be properly assembled into OXPHOS complexes and are rapidly degraded. The authors suggested that during translation, MRPL36 interacts with the ribosome at an as-yet unidentified step, which could be a critical for the production of assembly competent products.

A negative feedback mechanism for the regulation of mitochondrial translation based on an interplay between the translation and m-AAA protease, the key component of the mitochondrial quality control system has also been proposed for yeast (Nolden et al., 2005). The m-AAA protease not only degrades misfolded or damaged proteins, but also processes the mitoribosomal protein MRPL32. This maturation seems to be required for mitochondrial translation since the synthesis of mitochondrially encoded proteins was substantially impaired in cells lacking the m-AAA protease. Under certain conditions, misfolded, non-native respiratory subunits can compete with MRPL32 for binding to the m-AAA protease and thereby hamper the MRPL32 processing, ribosome assembly, and mitochondrial translation. MRPL32 processing and mitochondrial translation resume when the level of the respiratory subunits decreases as a consequence of their m-AAA-mediated degradation. It is worth mentioning in this context that plant m-AAA is able to process the yeast ribosomal protein MRPL32 (Kolodziejczak et al., 2002; Piechota et al., 2010). However, no direct evidence has been provided that plant m-AAA proteases can process a plant homolog of this mitoribosomal protein.

Given the mammalian mitochondrial transcripts contain no or only a short untranslated regulatory 5′ region (Koc and Spremulli, 2003), it seems unlikely that in mammals the synthesis of mitochondrial OXPHOS subunits is subject to CES, as it is in yeast. Instead, some reports indicate that members of the PPR protein family function as translational regulators of OXPHOS biogenesis in mammalian cells. One of them is leucine-rich PPR containing (LRPPRC) protein reported not only to coordinate mitochondrial translation but also to stabilize mitochondrial mRNAs and promote their polyadenylation (Sasarman et al., 2010). In the absence of LRPPRC the translation pattern is misregulated, with excessive translation of some transcripts and no translation of others. Interestingly, the two homologs of LRPPRC protein in fission yeast, Ppr4, and Ppr5, that also affect translation, play opposite roles in the expression of the *cox1* transcript (Kühl et al., 2011). Ppr4 specifically activates the translation of the *cox1* mRNA, whereas Ppr5 is a general negative regulator of mitochondrial translation. Furthermore, in humans another PPR protein – PTCD1 has been proposed to inhibit mitochondrial translation probably by decreasing the stability of the mitochondrial leucine tRNAs (Rackham et al., 2009). Other noteworthy human PPR proteins implicated in translation include PTCD2 involved in the maturation of cytochrome *b *transcripts (Xu et al., 2008) and PTCD3 associated with the SSU of mitochondrial ribosomes by binding 12S rRNA and necessary for efficient mitochondrial translation (Davies et al., 2009).

## CONCLUDING REMARKS

In bacteria mRNA abundance is the primary factor controlling the rate of protein synthesis. Recent studies have shown that in plant mitochondria the synthesis of OXPHOS subunits is surprisingly insensitive to the accumulation of respective transcripts (Kwaśniak et al., 2013; Wydro et al., 2013). It seems that, similarly to the chloroplast system (Marin-Navarro et al., 2007), the expression of mitochondrial genes in plants is mainly regulated at the level of translation. Compared with transcriptional regulation, translational regulation combined with selective degradation allows the organelles to modulate protein abundance more rapidly.

## Conflict of Interest Statement

The authors declare that the research was conducted in the absence of any commercial or financial relationships that could be construed as a potential conflict of interest.
